# The Effects of Gender and Self-Insight on Early Semantic Processing

**DOI:** 10.1371/journal.pone.0114421

**Published:** 2014-12-29

**Authors:** Xu Xu, Chunyan Kang, Taomei Guo

**Affiliations:** 1 School of Behavioral Sciences and Education, Pennsylvania State University at Harrisburg, Middletown, Pennsylvania, United States of America; 2 State Key Laboratory of Cognitive Neuroscience and Learning & IDG/McGovern Institute for Brain Research, Beijing Normal University, Beijing, People's Republic of China; 3 Center for Collaboration and Innovation in Brain and Learning Sciences, Beijing Normal University, Beijing, People's Republic of China; University of Leicester, United Kingdom

## Abstract

This event-related potential (ERP) study explored individual differences associated with gender and level of self-insight in early semantic processing. Forty-eight Chinese native speakers completed a semantic judgment task with three different categories of words: abstract neutral words (e.g., logic, effect), concrete neutral words (e.g., teapot, table), and emotion words (e.g., despair, guilt). They then assessed their levels of self-insight. Results showed that women engaged in greater processing than did men. Gender differences also manifested in the relationship between level of self-insight and word processing. For women, level of self-insight was associated with level of semantic activation for emotion words and abstract neutral words, but not for concrete neutral words. For men, level of self-insight was related to processing speed, particularly in response to abstract and concrete neutral words. These findings provide electrophysiological evidence for the effects of gender and self-insight on semantic processing and highlight the need to take into consideration subject variables in related research.

## Introduction

Research comparing abstract words (e.g., effect) and concrete words (e.g., table) has been abundant. The concreteness effect is often revealed, whereby people perform better with concrete words than with abstract words on a variety of tasks such as memory recall, lexical decision, sentence processing, and translation [1]–[6]. In recent years, another semantic feature, emotionality, has received much attention. Studies have shown that emotionally arousing words demonstrate processing advantage over neutral words [7].

Research on concept representation offers perspective on the distinctions between abstract, concrete and emotion words. A concrete concept, such as table or television, usually has a core referent, whereas an abstract concept, such as effect or consultation, is usually represented as a situation or a scenario with a number of key elements [8]. Among these elements, subjective experience, i.e., what one thinks and how one feels in a certain situation, is essential for the representation of abstract concepts [9]. For example, the representation of consultation should involve an agent with a motive of seeking information, and possibly also a feeling of eagerness. Therefore, one's own experience, direct or indirect, with such situation as well as one's understanding about this experience must be an integral part of forming and developing the proper concept. Just as Barsalou pointed out, introspection plays an important role in the representation of abstract concepts [10]. In this regard, emotion concepts (e.g., joy, guilt, sadness, and excitement) may be considered an extreme case, the formation and development of which rely primarily on introspective effort.

People reliably differ in their levels of self-insight, i.e., awareness and understanding of one's subjective experiences including cognitive or affective states and processes [11]. Consider the aforementioned representational differences between concrete concepts and abstract concepts, including emotion concepts as the special case in the abstract domain, individual variations in self-insight might be reflected as differences in semantic knowledge about abstract words in general and emotion words in particular. Specifically, a high level of self-insight should be reflected as rich semantic knowledge about words that denote abstract concepts involving cognitive or affective experiences and particularly about words that denote complex emotions, which may eventually translate into speedy and effortless processing of these words. Intuitively, introspective clarity or insight may not be as relevant to the processing of concrete words. However, concept formation or word learning in general requires reflecting upon one's own perception, memory, and thought processes. For example, car is an obvious concrete word, but driving experience must be part of our knowledge about cars. In addition, the process of learning to read, to write, and to understand the meaning of this word is also an experience subjective to introspection. Consequently, efficient processing of concrete words might also relate to one's level of insight. It thus can be speculated that self-insight is relevant to word processing in general, but the extent to which it is relevant may vary across different word categories, depending on how much introspective knowledge is involved in acquiring the concept.

The present study utilized the ERP technique to examine the effect of self-insight on semantic processing for three categories of words: abstract neutral words (e.g., logic and effect), concrete neutral words (e.g., teapot and table), and emotion words (e.g., despair and guilt). It was expected to reveal individual differences in word processing, which were associated with different levels of self-insight. After an extensive review of the literature about emotional words, Citron indicated that research on individual differences, particularly among healthy population, has been lacking [7]. In fact, individual differences in semantic processing in general are very much in need of investigation. This is especially true when it comes to words denoting abstract entities or events. This study was therefore expected to contribute to the literature along these lines. Furthermore, through examination of the relations between self-insight and ERP responses to different word categories, findings of this study may reveal potential electrophysiological evidence for representational differences across word categories.

Structural and functional differences between the male brain and the female brain, including the sexually dimorphic nature of the amygdala, have been well documented [12]. Gender thus appears particularly relevant to the aim of the present study to examine individual differences across and within different word categories, including emotion words. In particular, converging evidence has clearly shown that women experience more self-conscious emotions such as guilt relative to men [13], which indicates that women and men may differ in their levels of introspective knowledge regarding at least some aspects of their affective experiences.

Gender is one of the few individual differences variables that have been investigated for semantic processing. However, research on gender differences along the time course of word processing is limited. Wirth et al. analyzed participants' N400 responses in a word priming task [14]. Female participants were found to always engage in more elaborative processing of semantic information than did male participants. Daltrozzo et al. obtained the same results in the N400 time window, but reported a lack of significant gender difference in P300 [15]. The reason for the lack of evidence for gender variation at the early stage could be due to possible insensitivity of the early ERP component. Alternatively, it could be due to the specific word samples chosen by the studies. As Daltrozzo et al. pointed out, men and women responded to different categories of words differently [15]. To achieve a certain level of balance across different semantic categories, their study incorporated various types of words including a small portion of abstract words and erotic words. Similarly, the study by Wirth et al. included 50% abstract words and 50% concrete words [14]. However, neither study conducted gender comparison within each of the different word categories.

A study by Sass et al. examined gender differences in early ERP components in response to pleasant words, threat words, and neutral words [16]. They found that men showed preferential processing of threat words at about 100 ms post word onset, while women showed preferential processing of threat words not until 300 ms post word onset. Extending from these earlier studies, the present study incorporated gender as another individual differences variable along with level of self-insight to investigate their effects on early processing of emotion words, abstract neutral words, and concrete neutral words. With these different word categories, the study was expected to extend the knowledge about the timing of gender variation in semantic processing across word categories and potential gender contrast in the relationship between self-insight and semantic knowledge.

In sum, this study aimed to examine the effects of gender and self-insight on the early stage of word processing with abstract neutral words, concrete neutral words, and emotion words. The study utilized a paradigm to obtain an early ERP component, the Recognition Potential (RP), which is considered an index for semantic activation, peaking between 200 and 250 ms [17], [18]. With a mixed word sample, gender difference in processing level has been shown emerging in the N400 time window [14], [15]. With emotionally arousing words, research found gender difference before 300 ms post stimuli [16]. It was therefore predicted that the RP would reveal gender difference in early processing of emotion words. However, Rudell and Hua showed that the RP was sensitive to individual differences in reading ability [19]. Therefore, should gender difference exist in early word processing in general, not just in the processing of emotion words, the RP would also reveal gender variation in response to abstract and concrete neutral words. In such case, women were expected to show a greater processing level relative to men [14], [15].

The investigation of the relationship between level of self-insight and word processing was exploratory in nature. Based on the above discussion that representations of abstract words and emotion words contain essentially introspective knowledge relative to that of concrete words, a tentative prediction was that level of self-insight would show stronger association with the processing of abstract words and emotion words than with the processing of concrete words. Gender variation might exist with regard to the relation between self-insight and word processing, particularly for emotion words given that women and men showed differential responses to this type of words at early processing stage and different levels of introspective knowledge about this type of concepts [13], [16].

As for RP differences across word categories, based on past research [20], [21], it was predicted that concrete neutral words would elicit greater RP than abstract neutral words. In addition, in one study with similar experimental paradigm, Hinojosa et al. found in the 225–300 ms time window greater negativity for some words with emotional significance relative to neutral words [22]. It was therefore predicted that emotion words might lead to greater RP than abstract and concrete neutral words.

Finally, one thing needed to be pointed out is that many studies on the effect of emotionality in word processing intermixed emotion words (e.g., despair, guilt, joy, sadness) that denote emotion states or processes with emotional words (e.g., success, hostility, flower, and enemy) that denote non-emotion concepts but evoke emotional responses [7]. Because emotional words can vary in abstractness, these studies often employed the factorial design to examine both abstractness and emotionality, and often reveal complex, yet inconsistent, interactions between the two factors [23]–[25]. Acknowledging complexity of the interplay between abstractness and emotionality in word processing, for the specific purpose of this research, the experiment described below utilized only emotion words in order to detect potential individual differences in the representation and processing of emotion concepts relative to abstract and concrete neutral concepts. As indicated by prior research on emotion words, they are abstract in nature [26], [27]. Therefore, the present study employed a one-way design to compare the three types of words.

## Method

### Ethics Statement

This study was approved by the Institutional Review Board of the Imaging Center for Brain Research of Beijing Normal University. All participants gave written informed consent prior to the experiment and were paid for their participation.

### Participants

Fifty-five college students were recruited for the experiment. The final analysis excluded five participants with signals from electrodes of interest interfered by unknown noise, another participant with a response accuracy rate lower than 75%, and yet another participant with no accurate responses due to misunderstanding of task instruction. This resulted in 48 participants (25 females) with an average age of 22.06 (*SD* = 1.82) ranging from 19 to 26. They were all native speakers of Chinese, right-handed, with normal or corrected to normal vision. As one of the recruitment criteria, right-handedness was determined based on participant report, and further verified by the experimenter through observation during the course of the experiment.

### Experimental Stimuli

Following the paradigm described by earlier studies of RP [20], the experimental stimuli included real words, non-words, and background stimuli. There were three types of real Chinese two-character words (all nouns): 48 abstract words, 48 concrete words, and 48 emotion words (28 negative words and 20 positive words). These real words were sampled from a database that contained 715 Chinese two-character words with normative information on frequency, part of speech, number of strokes, and emotionality ratings. The emotionality was assessed using a 5.0 scale (0 =  “neutral” and 4 =  “high emotional value”). For the purpose of this study, abstractness ratings were collected using a 6.0 scale (1 =  “highly concrete” and 6 =  “highly abstract”) from a group of 21 Chinese native speakers who did not participate in the main experiment. Considering that this rating process can be sometimes difficult, an even number (6.0) was chosen for this bipolar scale (highly concrete versus highly abstract) in order to prevent potentially a large number of convenient responses by selecting the mid-point of the scale (e.g., 4 on a 7.0 scale). The properties of the sampled abstract, concrete and emotion words are displayed in Table 1. The three types of words were matched on frequency (*F*<1) and number of strokes (*F* = 2.30, *p*>.10). Abstract words and concrete words were also matched on emotionality ratings (*p*>.45), both rated lower than emotion words (both *p*s<.001). Abstract words and emotion words were matched on abstractness ratings (*p*>.27), both rated higher than concrete words (both *p*s<.001). In addition, to constitute a semantic judgment task, 48 animal words were collected with their numbers of strokes matched with the three types of real words.

**Table 1:** Properties of the three types of real words.

Property	Word Type	Mean	SD
frequency (log)	abstract words	3.03	1.50
	concrete words	2.84	1.24
	emotion words	3.02	1.20
number of strokes	abstract words	16.75	3.39
	concrete words	17.98	4.23
	emotion words	18.42	4.18
emotionality rating	abstract words	0.16	0.11
	concrete words	0.10	0.11
	emotion words	2.40	0.36
abstractness rating	abstract words	4.07	0.56
	concrete words	1.46	0.31
	emotion words	4.20	0.23

There were 48 non-words. A non-word was created by cutting two Chinese characters each into half and then re-arranging the four pieces. As a result, these non-words appeared to resemble real two-character words, but were devoid of meaning. There were 240 background stimuli. A background stimulus was created by cutting a pair of two Chinese characters into 32 pieces and re-arranging them randomly to form an image without word-like characteristics or meanings. Exemplars for each type of stimuli are listed in the S1 Appendix.

### Measure for Level of Self-insight

The Self-Reflection and Insight Scale (SRIS) [11] served to assess individual differences in self-insight. It contains 20 statements. Respondents indicate how much each statement describes their own characteristics by rating on a 6.0 scale. The SRIS consists of two subscales, self-reflection (SRIS-SR) and insight (SRIS-IN). SRIS-SR was designed to measure “the inspection and evaluation of one's thoughts, feelings, and behaviors,” while the SRIS-IN was intended to measure “the clarity of understanding of one's thoughts, feelings, and behaviors” [11]. Grant et al. indicated that scores from SRIS-SR were not always positively associated with, and thus not reflective of, the level of insight [11]. Therefore, for the purpose of this study, only the summation score collected from SRIS-IN was used to assess individual differences in self-insight. In this study, participants' insight scores ranged from 15 to 45, with a mean of 32.17 (*SD* = 6.07). There was not a significant difference between men (*M* = 33.17, *SD* = 6.56) and women (*M* = 31.24, *SD* = 5.56), *p*>.27.

### Procedure

All participants signed a written informed consent form prior to the experiment. The experiment was carried out in a soft-lighted and soundproof recording room. All stimuli were presented white-on-black, 3.5 cm high and 6.6 cm wide, in the middle of the computer screen. Participants sat about 100 cm from the screen.

The Rapid Stream Stimulation paradigm [28], [29] was employed with a stimulus onset asynchrony (SOA) of 250 ms. The screen refresh rate was set at 60 Hz, and each stimulus was presented for 250 ms. The experiment included three sessions. Each session contained 16 abstract words, 16 concrete words, 16 emotion words, 16 animal words, 16 non-words, and 80 background images. Each session began with a string of asterisks (******), where participants were instructed that they could blink as much as they needed, and that they could begin the presentation of the stimuli by pressing a key when they were ready. Then, there were eight sequences in a session. Each sequence contained ten test stimuli, including two abstract words, two concrete words, two emotion words, two animal words, and two non-words presented in random order. Each word or non-word was preceded by four to six background stimuli. The presentation order of the sequences within each session was randomized, so was the presentation order of the sessions. Participants were instructed to press a key as quickly and accurately as possible whenever they detected an animal word. All stimuli were presented only once. During the experiment, there were two breaks, with duration determined by the participants.

To ensure that the participants understood the task, they practiced with a different set of stimuli before the experiment. After the experiment, they filled out the Self-Reflection and Insight Scale [11]. The whole experiment lasted about 1.5 hours.

### EEG Recording and Data Analysis

Participants' electroencephalograms (EEG) were recorded from a 32-channel Quik-cap (NeuroScan, Inc.) with the right mastoid as reference. Vertical eye movements were recorded by two electrodes symmetrically placed above and below the left eye. Horizontal eye movements were monitored by two electrodes placed on the left and right outer canthi. Impedances of all electrodes were kept below 5 kΩ. The sample rate was 500 Hz with a band-pass of 0.05–100 Hz. The data were re-referenced offline using the global average reference method, which was proved to be the best way to obtain the RP [20].

The continuous data were segmented from 100 ms pre-stimulus to 600 ms post-stimulus for abstract words, concrete words, emotion words, and non-words. Data were filtered offline with a low pass of 30 Hz (24 dB). The mean voltage of the 100 ms pre-stimulus interval acted as the baseline for ERP measurement. Wrong trials and trials contaminated by eye blinks, eye movements, or muscle potentials exceeding ±100 µV at any scalp electrode were excluded from analysis. Thus, about 6.66% of trials were excluded. The segmented data were then averaged for each word type within each participant.

Data extraction and analyses focused on average recordings from P7, P8, O1, and O2 electrodes, the areas where RP was found most evident [20], [28], [30]–[32]. The peak latency and peak amplitude value of RP were measured in the 150–400 ms time window that covered the presentation time of one stimulus and included the most negative peak of RP [19], [20]. More specifically, for each participant, a peak value and the corresponding peak latency were extracted from the most negative peak within the time window under each word condition at each of the four electrode sites.

To examine gender difference in RP peak latency across different word conditions, a mixed 4×2 ANOVA with word condition (abstract, concrete, emotion, and non-word) and gender as factors was conducted on RP latency at each of the four electrode sites of interest: P7, P8, O1, and O2. The same analysis was applied to examine gender difference in RP peak amplitude. For the above analyses, the Geisser-Greenhouse correction for non-sphericity was applied when appropriate, with unadjusted degrees of freedom presented. To examine the relationship between insight level and the RP, correlational analysis was first performed. Regression analysis then further examined the role of self-insight in semantic activation of real words by partialing out individual variation in response to non-words.

## Results

### Gender Differences

#### Latency

**Figure 1:**
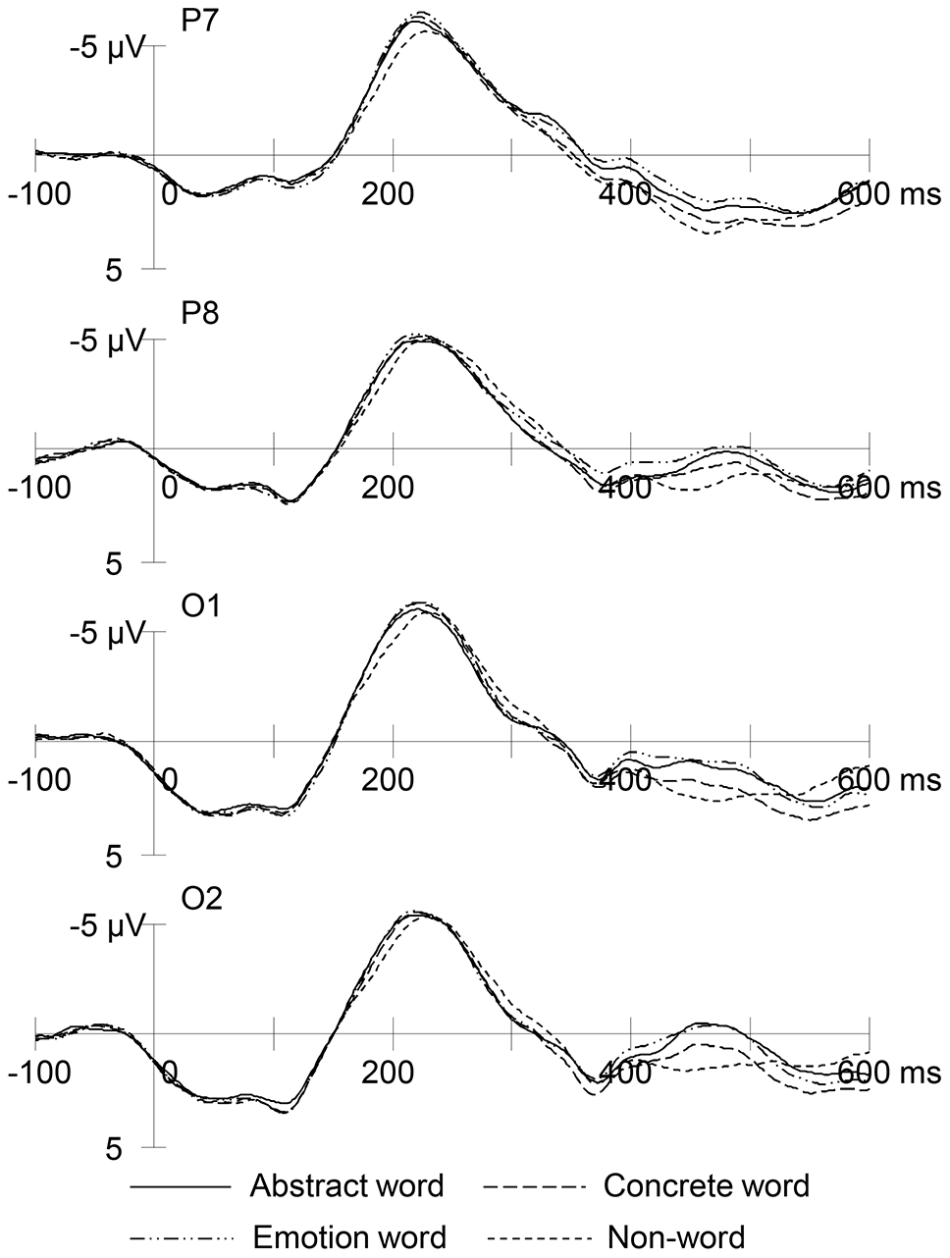
Grand mean RP waveforms in response to abstract words, concrete words, emotion words, and non-words at four electrode sites.

Fig. 1 presents the RP at the four chosen electrode sites. The mixed 4×2 ANOVA with word condition and gender as factors revealed a significant word condition effect only at P8, *F* (3, 138)  = 7.42, *p*<.001. The same effect seemed to approach significance at P7 and O2, both *p*s = .07. Bonferroni *t*-tests showed that, at P8, abstract words (*M* = 225 ms) and emotion words (*M* = 220 ms), but not concrete words, elicited shorter RP latency than did non-words (*M* = 234 ms), both *p*s<.04. In addition, emotion words (*M* = 220 ms) had shorter RP latency than concrete words (*M* = 227 ms), *p* = .02. No gender effect or gender by word interaction approached significance at any sites.

#### Peak amplitude

**Figure 2:**
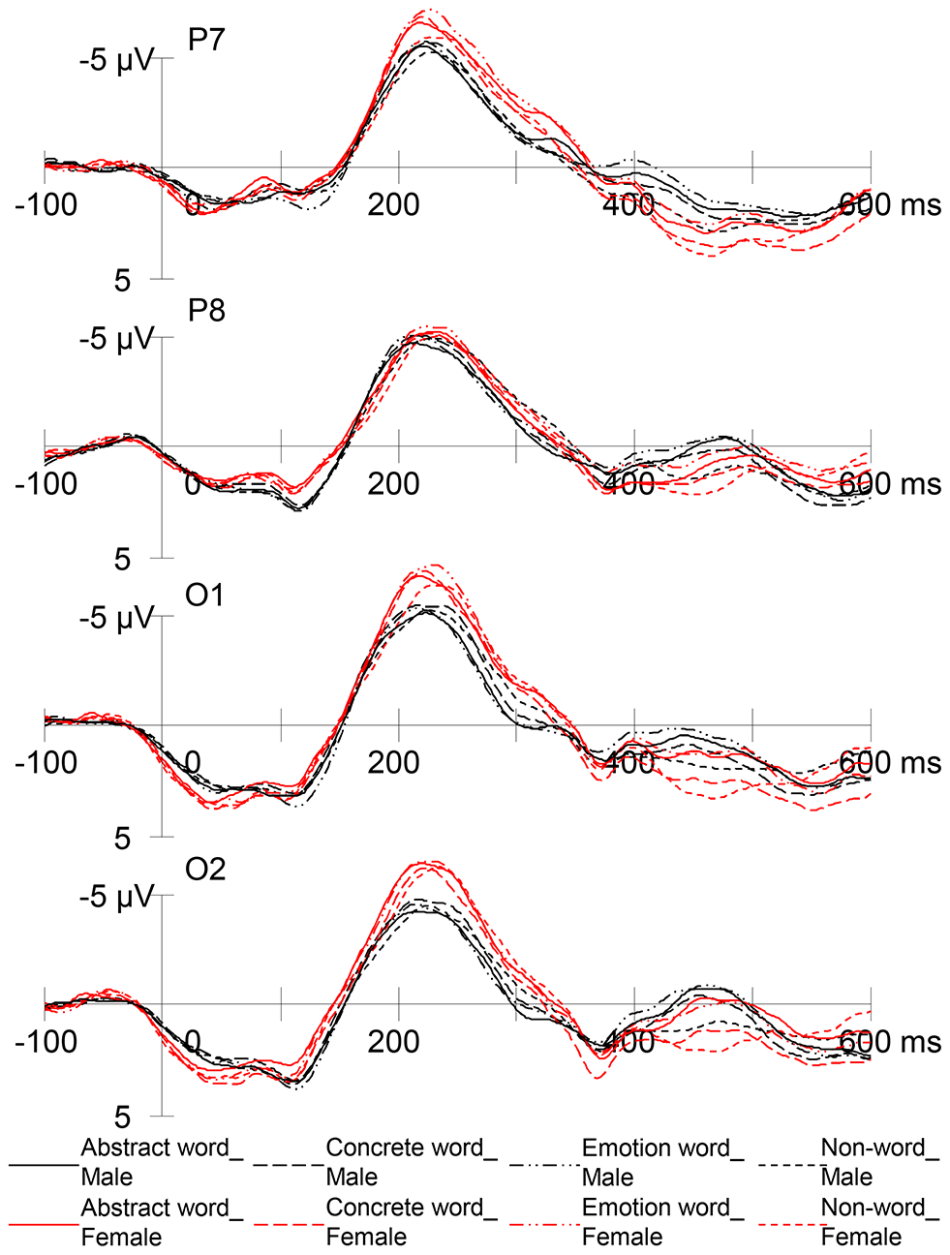
Grand mean RP waveforms of male and female participants in response to abstract words, concrete words, emotion words, and non-words at four electrode sites.

To examine potential gender difference in level of processing, the 4×2 mixed ANOVA with word condition and gender as factors was applied to RP peak amplitude at P7, P8, O1, and O2, respectively. Fig. 2 contrasts the RP between two gender groups. At O2, female participants (*M* = −7.63 µV) showed significantly greater RP than male participants (*M* = −6.04 µV), *F* (1, 46)  = 4.72, *p* = .035. The absence of gender by word interaction indicated that this difference was consistent for all real word conditions and the non-word condition, which may suggest that, compared to men, women engage in deeper processing in response to both written words and word-like visual stimuli.

The ANOVA also revealed a significant main effect of word condition at P7, *F* (3, 138)  = 12.06, *p*<.0000005. Bonferroni *t*-tests showed that all three real word conditions (*M* = −7.22 µV for abstract words, *M* = −7.39 µV for concrete words, and *M* = −7.55 µV for emotion words) led to significantly greater RP than did the non-word condition (*M* = −6.55 µV), all *p*s<.005, which indicated that semantic activation was evident at P7. No other effects appeared significant at this or other sites.

### Level of Self-insight

**Figure 3:**
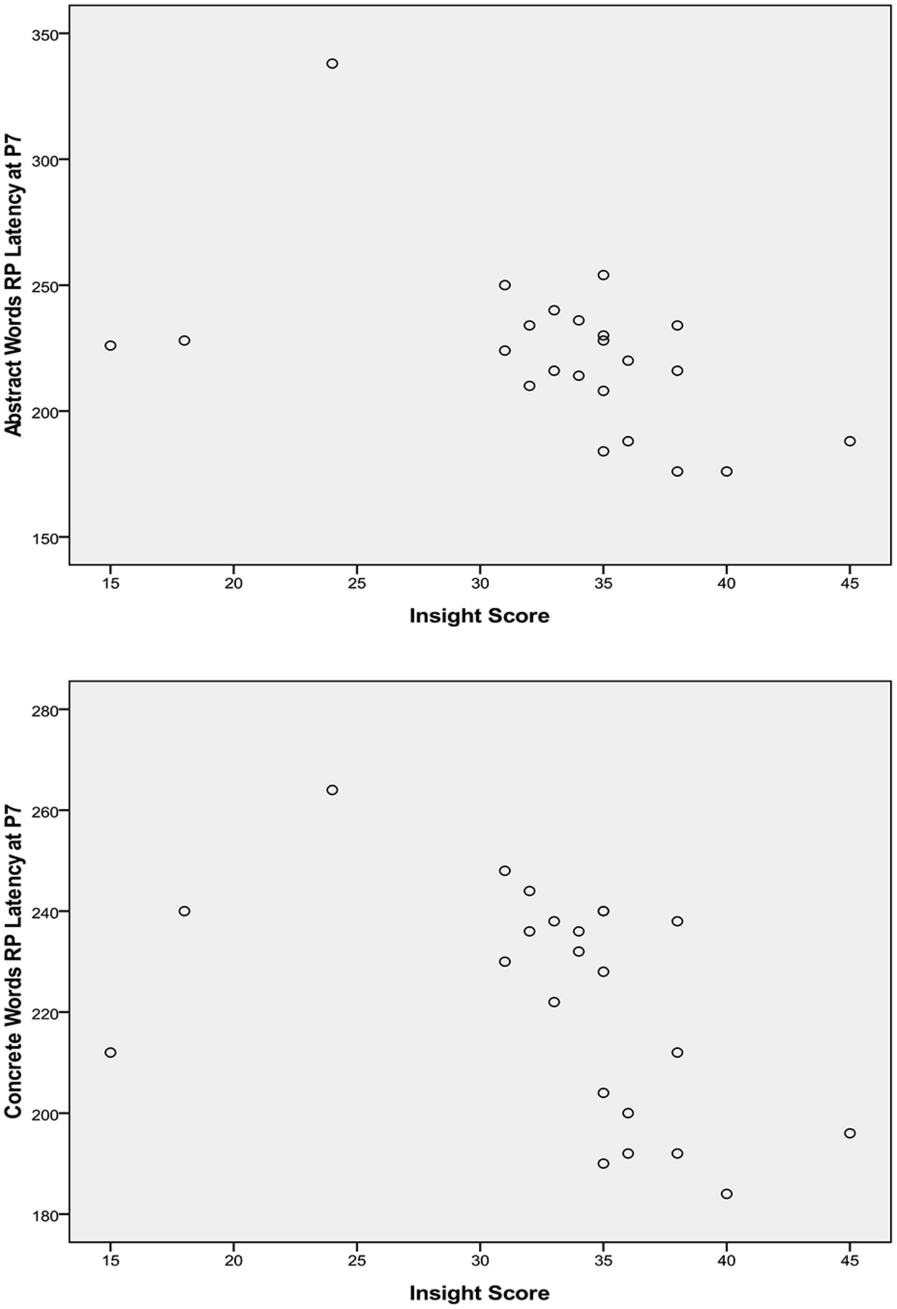
Correlation of insight scores and male RP peak latency at P7 in response to abstract words and concrete words.

**Figure 4:**
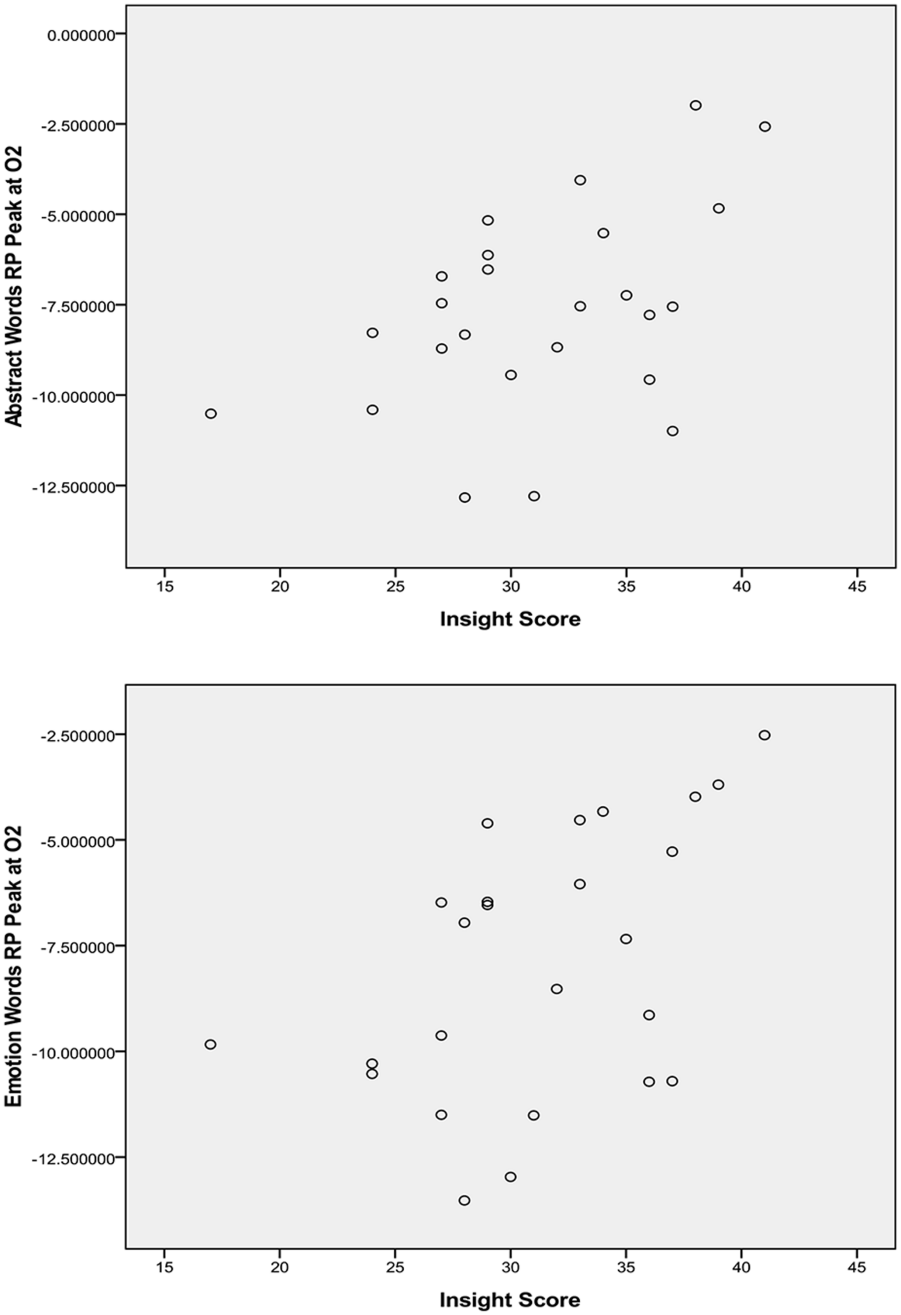
Correlation of insight scores and female RP peak amplitude at O2 in response to abstract words and emotion words.

To reveal potential gender contrast in the relationship between self-insight and word processing, correlational analysis was first performed for each gender group. For the male group, insight scores appeared negatively related to RP latency at all sites, but most evidently at P7 for abstract words, *r* (23)  = −.478, *p* = .021, and concrete words, *r* (23)  = −.441, *p* = .035. Higher insight scores were associated with shorter latency for both types of words (Fig. 3). For the female group, insight scores showed a significant positive correlation with RP peak amplitude at O2 for abstract words, *r* (25)  = .461, *p* = .020, and emotion words, *r* (25)  = .482, *p* = .015. Lower insight scores were associated with greater RP for both types of words (Fig. 4).

To test whether insight level accounts for variation in semantic activation instead of simply variation in RP responses to word and non-word stimuli in general, regression analysis was applied to further examine the effect of insight scores on RP responses to the real words identified in the above correlational analysis. Specifically, for the male group, RP latency at P7 in response to abstract and concrete words was regressed upon insight score after partialing out variation in RP latency in response to non-words at this electrode site. After controlling for RP latency of non-words, insight scores no longer accounted for a significant amount of variation in RP latency for abstract words (Δ*R^2^* = .07, *F* (1, 20)  = 3.14, *p* = .09) or concrete words (Δ*R^2^* = .05, *F* (1, 20)  = 2.21, *p* = .15).

For the female group, RP peak amplitude at O2 in response to abstract and emotion words was regressed upon insight score after partialing out variation in RP peak amplitude in response to non-words at O2. After controlling for variation in non-words, insight scores still accounted for a significant amount of variation in RP peak amplitude for abstract words (Δ*R^2^* = .07, *F* (1, 22)  = 8.34, *p* = .009), and for emotion words (Δ*R^2^* = .10, *F* (1, 22)  = 7.17, *p* = .014).

## Discussion

Through examining the RP, an early ERP component, during a semantic judgment task, this study investigated the effects of two individual differences variables, gender and level of self-insight, on early semantic processing. The experiment utilized emotion words, abstract neutral words, and concrete neutral words. Results indicated that women showed a greater level of processing, as assessed by RP amplitude, in response to real word and word-like stimuli than did men. Level of self-insight demonstrated differential relations with RP responses of men versus women. For men, the effect of self-insight manifested as difference in speed of processing, whereas for women, the effect manifested as difference in level of processing. Most notably, for women, level of self-insight accounted for a significant amount of variation in semantic activation for abstract neutral words and emotion words.

### Individual Differences

Early research has shown that women tend to engage in more elaborative semantic processing [14], [15], [33]. However, with a mixed word sample, gender difference did not become evident until the N400 time window [15]. This study found that, as early as 200–250 ms post stimuli, female participants showed greater electrophysiological activities than male participants in response to real word and word-like visual stimuli. Further, gender difference did not just manifest at the early stage of processing for semantically salient emotion words, as shown by Sass et al. [16]. It was evident across three word categories. The RP is considered an index representing the process of analyzing visual form to gain semantic access [17]. Therefore, gender difference revealed here in both real words and non-words suggests that women start to engage in deeper processing than men not only before semantic integration as indexed by N400 in previous studies, but also before actual semantic access is attained. In the literature, the time course and mechanism of verbal processing by which the male brain and the female brain differ have not been fully investigated [14]. The findings of this study add to the literature with regard to the timing of gender differences in semantic processing. It awaits future research to further investigate whether such early gender variations manifest specifically in response to word-like visual stimuli, and what characteristics of visual stimuli trigger such differential reactions.

**Figure 5:**
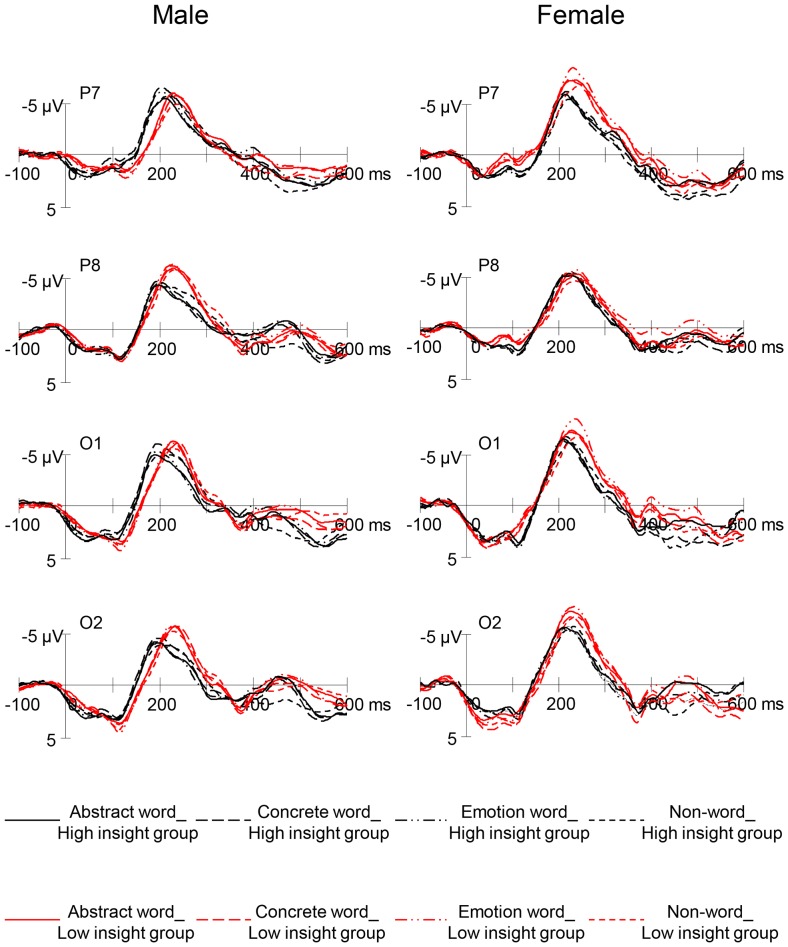
Differential relations between insight and RP waveforms for male versus female participants.

Gender differences were further evidenced in the relationship between level of self-insight and semantic processing. This finding was astonishing in that the two gender groups were qualitatively different in terms of how level of self-insight influenced the RP waveform. To better depict this contrast between the female and the male participants, within each gender group, a median split based on their insight scores divided participants further into two groups, the high insight group and the low insight group. Fig. 5 displays RP responses by the high and low insight groups for male versus female participants.

For the female group, at the electrode site O2, higher level of self-insight was found significantly associated with smaller RP amplitude in response to abstract words (e.g., logic, feature, and tradition) and emotion words (e.g., despair, sadness, and gratitude). This was true even after controlling for individual variation in response to non-word visual stimuli. That is, level of self-insight can explain a significant amount of variation in the level of semantic activation for abstract and emotion words. Individuals who reported lower levels of self-insight showed enhanced activation to these two types of words, whereas those who reported higher levels of self-insight showed relatively reduced activation to these words. Statistically, this relationship did not hold for the processing of concrete words (e.g., teapot, window, and vehicle). The differential relationship of insight to the processing of different categories of words may be a reflection that the level of introspective knowledge involved in word processing varies as a function of these word categories. As discussed earlier, research has indicated that subjective experiences are an integral part of the representation of abstract concepts [9]. Intuitively, this seems particularly the case for the representation of emotion concepts, whereas the representation of concrete concepts may rely to a much lesser degree on introspective knowledge about one's subjective experiences. Recent research [34], [35] on abstract concepts seems to offer even further explanation for the parallel between abstract and emotion words revealed by the present study. These studies have shown that one's emotional experiences play a crucial role in the representation of abstract concepts. That is, involvement of emotional experiences, rather than other types of subjective experiences, in the representations of abstract and emotion words could be the primary reason why processing both types of words were found closely related to self-insight. The finding that insight appeared more relevant to the activation of abstract words and emotion words relative to concrete words therefore may be considered further evidence for the representational differences across word categories. However, it is not clear why this was only reflected in the RP of female participants. It might be possible that the greater RP of this gender group rendered the relationship between insight and processing level more detectable. This certainly requires further research to be addressed.

The revealed association of insight with the processing of abstract and emotion words among women was correlational in nature, and it needs future research to explore potential causality. On one hand, it is possible that an inherent self-perception of uncertainty about one's own subjective experiences results in more extensive activation of introspective knowledge during processing of these words, and hence an augmented level of electrophysiological reaction. On the other hand, it is also possible that a predisposed tendency to engage in enhanced electrophysiological reactions to words that evoke introspective knowledge leads to a self-perception of uncertainty about relevant experiences. More likely the two factors are intertwined during the course of mental development. Research on the relationship between these factors may have implications for intervention with cognitive and affective disorders.

For the male group, the relationship between insight level and RP responses to word processing appeared less conclusive. In contrast to the female group, a higher level of self-insight was found associated with shorter RP latency, particularly evident in response to abstract and concrete words at the electrode site P7. However, insight scores did not account for latency variation in response to these real words above and beyond individual variation in response to non-word visual stimuli. This may indicate that level of self-insight is not a reliable predictor for latency of semantic activation. Alternatively, this may be partially due to the fact that insight scores among male participants appeared negatively skewed. While the majority of the male participants' scores were well above the mid-point of the insight scale, three participants reported substantially lower insight scores (Fig. 3), which may or may not be an unbiased reflection of their actual insight levels. Analysis excluding these three participants revealed even stronger correlation between insight and RP latencies for these words. While subjectivity is inherent to self-report measures, a larger sample may help elucidate the relationship of insight and processing speed among male participants in future research.

For male participants, the finding that higher insight tended to be associated with shorter latency in response to word stimuli (Pearson's *r*s ranging from.33 to.48 for emotion, concrete, and abstract words) may prompt one to ponder whether language proficiency, particularly vocabulary knowledge, is the underlying factor for the relationship between insight and the speed of semantic judgment. Rudell and Hua indeed reported an association between RP and language proficiency as assessed by GRE verbal test [19]. Their participants in the fourth quartile of GRE verbal scores generated the longest RP latency and the smallest RP amplitude. This association appeared to hold for both men and women. However, in the present study, low insight scores were associated with longer RP latency only for the male group. For the female group, low insight scores were associated with greater RP amplitude. This contrast suggests that verbal proficiency and insight are two factors that, though possibly partially overlapping, differ in the mechanisms by which they influence the RP waveform. Future research is needed to investigate the mechanism through which insight, gender, and verbal proficiency interact in the process of language processing.

### Different Word Categories

This study found that the three real word categories elicited comparable levels of RP. The lack of difference between abstract words and concrete words was in contrast to Martin-Loeches et al. [20], which utilized the same experimental paradigm and revealed that concrete words elicited greater RP than abstract words. The following might have contributed to this discrepancy. First, the present study included emotion words as a separate category of comparison, and excluded emotion words from the abstract word category, which resulted in a word sample with different semantic features from that of the earlier study. Further, to distance abstract and concrete words from emotion words, the emotionality ratings of these two categories of words were designed to fall on the lower end of the scale. Under this constraint, matching emotionality, frequency, and stroke number between abstract and concrete conditions further restricted word sampling. As a result, the contrast in abstractness ratings between the two conditions was relatively limited compared with the rating contrast in the earlier study. A numerical comparison of rating difference between the two studies showed that this was indeed the case. Second, the fact that the two studies were conducted in two different languages, Spanish and Mandarin, might also have been a factor. Specifically, non-words in the present study contained recognizable radicals and thus, to a large degree, preserved word-like visual features. A more careful control for visual form similarity between abstract words and concrete words relative to non-words may help to detect semantic processing differences between these two real word conditions. Further research is necessary to investigate RP amplitude in response to the processing of different semantic features as well as the application of RP paradigm to research in different languages.

Finally, emotion words did not elicit greater RP than did abstract and concrete words. In the literature about word emotionality, more studies have been done through examining another early ERP component, the early posterior negativity (EPN). Some studies reported that the EPN was amplified by emotionally significant words around 250 ms post word onset [22], [25], [36]. However, other studies using emotional words did not find the EPN [23], [24]. The EPN has been thought to signify allocation of attentional resources [37], [38], and functionally linked to the RP [39]. Therefore, the lack of difference between emotion words and neutral words in the present study could be due to the semantic judgment task, which might to some extent have diverted attention away from word emotionality. Further, the fact that the present study utilized only emotion words (not emotionally significant words) might also have been a factor. Unfortunately, there does not seem to be research looking into processing differences between words that denote specific emotion states or processes and words that have general emotional connotations. A follow up study seems appropriate to address this issue.

### Limitations

As emphasized earlier, the RP is an early ERP component indexing early stage of word processing. The findings of this study likely reflect mainly the automatic and implicit stage of semantic processing. To gain a more comprehensive understanding about processing differences across various word categories and individual differences in word processing, later ERP components certainly need to be investigated. The Rapid Stream Stimulation (RSS) paradigm in this study is generally utilized to enhance the RP [29]. A different design is needed to obtain more standard later ERP components, which will be adopted in future research to examine the more controlled and explicit stage of semantic processing.

This study employed a semantic judgment task with animal words serving as the target words to be responded to by participants. There might be a possibility that this design could have distorted the differences between abstract, concrete, and emotion words. More specifically, animal words denote a type of concrete concept that might share greater conceptual similarities with concepts denoted by concrete words than those denoted by abstract words or emotion words. Consequently, word processing during the experiment might have been biased toward concrete words, but away from abstract and emotion words. It is not clear how much difference this could make at the early stage of word processing. However, for future research, a design to induce more balanced processing across word categories would be helpful.

Lastly, the primary goal of this study was to explore individual differences in word processing. Although emotion words were included as one of the word categories for investigation, valence was not taken into consideration. Roughly half positive words (e.g., joy and excitement) and half negative words (e.g., guilt and despair) constituted the category of emotion words. In the literature of word emotionality, valence has been shown a complex factor as evidenced by inconsistent reports with regard to its modulations of emotionally sensitive ERP components such as the EPN and the late positive component [22]–[25]. Therefore, it should be acknowledged that separate investigations of positive emotion words and negative emotion words would help to enhance our understanding about processing differences across word categories, and possibly to reveal further individual variations.

## Conclusions

This study found gender difference in the level of early stage semantic processing. In addition, level of self-insight exhibited a differential relationship with word processing for men versus women. Generally speaking, for men, high insight appeared to be associated with a greater processing speed. For women, low insight appeared to be associated with a greater processing level. These relations were found to vary as a function of word categories with different semantic features, e.g., concreteness and emotionality, which may reflect representational differences across these word categories. Although many questions remain to be answered about the mechanism by which gender, self-insight, and semantic knowledge interact in word processing, the individual variations revealed in this study highlight the need to take into consideration subject variables when utilizing electrophysiological measures in related research.

## Acknowledgments

The authors would like to thank Wuwei Fan, Jie Lin, and Haoyun Zhang for their assistance with data collection. We also sincerely thank Kathy Brode and Christopher Twilley for proofreading this manuscript.

## Supporting Information

S1 Appendix **Exemplar words for each type of experimental stimuli.** The abstract word shown here means logic in English, the concrete word teapot, the emotion word despair, and the animal word frog.
(TIF)
